# A Quantitative Metric to Identify Critical Elements within Seafood Supply Networks

**DOI:** 10.1371/journal.pone.0091833

**Published:** 2014-03-14

**Authors:** Éva E. Plagányi, Ingrid van Putten, Olivier Thébaud, Alistair J. Hobday, James Innes, Lilly Lim-Camacho, Ana Norman-López, Rodrigo H. Bustamante, Anna Farmery, Aysha Fleming, Stewart Frusher, Bridget Green, Eriko Hoshino, Sarah Jennings, Gretta Pecl, Sean Pascoe, Peggy Schrobback, Linda Thomas

**Affiliations:** 1 Climate Adaptation Flagship, Commonwealth Scientific and Industrial Research Organisation, Brisbane, Queensland, Australia; 2 Climate Adaptation Flagship, Commonwealth Scientific and Industrial Research Organisation, Hobart, Tasmania, Australia; 3 University of Tasmania, Hobart, Tasmania, Australia; 4 Queensland University of Technology, Brisbane, Queensland, Australia; Leibniz Center for Tropical Marine Ecology, Germany

## Abstract

A theoretical basis is required for comparing key features and critical elements in wild fisheries and aquaculture supply chains under a changing climate. Here we develop a new quantitative metric that is analogous to indices used to analyse food-webs and identify key species. The Supply Chain Index (SCI) identifies critical elements as those elements with large throughput rates, as well as greater connectivity. The sum of the scores for a supply chain provides a single metric that roughly captures both the resilience and connectedness of a supply chain. Standardised scores can facilitate cross-comparisons both under current conditions as well as under a changing climate. Identification of key elements along the supply chain may assist in informing adaptation strategies to reduce anticipated future risks posed by climate change. The SCI also provides information on the relative stability of different supply chains based on whether there is a fairly even spread in the individual scores of the top few key elements, compared with a more critical dependence on a few key individual supply chain elements. We use as a case study the Australian southern rock lobster *Jasus edwardsii* fishery, which is challenged by a number of climate change drivers such as impacts on recruitment and growth due to changes in large-scale and local oceanographic features. The SCI identifies airports, processors and Chinese consumers as the key elements in the lobster supply chain that merit attention to enhance stability and potentially enable growth. We also apply the index to an additional four real-world Australian commercial fishery and two aquaculture industry supply chains to highlight the utility of a systematic method for describing supply chains. Overall, our simple methodological approach to empirically-based supply chain research provides an objective method for comparing the resilience of supply chains and highlighting components that may be critical.

## Introduction

Supply chains describe the multitude of processes and activities that connect products and services with consumers [1]. Rather than being linear, supply chains typically take the form of networks of nodes with upstream and downstream linkages, analogous to ecological networks that describe the flow of biological matter from primary producers to top predators. Empirically, supply networks [2] vary depending on the number of components (i.e. processes and activities) and how vertically integrated they are (fewer steps in the chain indicate a more vertically integrated supply chain), how many product forms are supplied to consumers and the number of markets supplied. As is often the case, we use the terms supply chain and supply network interchangeably. Supply chains generally refer to a simpler, linear system with a unidirectional flow of goods or services, while supply networks generally involve a more complex chain with lateral links, reverse loops and two way exchanges. Systematic methods for describing supply chains can assist in understanding and comparing network properties, as well as identifying strengths and weaknesses in supply chains [3]. In the first instance, most supply chains can be organised into typical components comprising primary production, processing, storage and transport, marketing (wholesale and retail) and the final consumer. Whole of network methods and simple metrics can be used to analyse individual chains and facilitate cross-comparisons with other systems. In particular, identification of key elements along the supply chain may assist in informing adaptation strategies to reduce anticipated future risks posed by climate change [4].

Prices are commonly used in the economic literature to describe relationships along the supply chain as the key interest lies in understanding how price changes due to supply and demand shocks at one level of the supply chain (e.g. production) are transmitted to other levels (e.g. wholesale, retail, final consumption). For example, the price transmission literature has largely focused on how asymmetry in price movements along the chain reflects differences in market power and/or differing ability to adjust to change [5], with analysis of marketing margins along the supply chain also used to provide information on market power [6], [7]. The market integration literature also uses prices to consider the degree of price transmission at different levels in the supply chain and across supply chains (i.e. by considering potentially competing products) [8], [9], [10].

When quantities are available, demand models provide additional information on the inter-relation between prices and quantities in markets as well as the effect that changes in consumer incomes, exchange rate, population and other factors can have on demand [11], [12], [13]. Hobday et al. [14] have built inverse demand models within a spatial equilibrium framework [15], [16] to simulate the effect that changes in supply (e.g., due to climate change), and the exchange rate can have on relative trade flows of lobster between two markets. Mullon et al. [17] additionally integrate ecological and economic dynamics within a bio-economic framework to simulate under different scenarios the consequences of both global and local changes in fishmeal and fish oil markets and their supply chains.

We propose a simple quantitative metric that can complement the economic, logistics and operations research literature and does not require time series data. Instead, discussions with stakeholder groups participating at different levels in the supply chain are used to establish the elements in the supply chain, how these are linked and the proportion of the product that moves through each element to identify the connectedness (or complexity) of the system. The SCI method also allows actual values to be used instead of proportions. Our Supply Chain Index (SCI) identifies critical elements as those elements with large throughput rates, as well as greater connectivity (i.e. more links in and out). We highlight the important role of connectance in determining the resilience of a supply chain to perturbations, such as those which would result from climate change. We use the term resilience rather than resistance, which implies an ability to carry on as before, because future climate changes are likely to be ongoing and uncertain, requiring whole supply chains to be more flexible and adaptable as shocks and challenges become more frequent and difficult. Moreover, the distribution of the SCI scores within a supply chain provides a novel perspective on the connectedness of a supply chain when compared with other examples from that sector, as illustrated for the fisheries and aquaculture examples we present, and a basis on which to discuss its potential resilience to change.

Drawing on the similarities between ecological networks and supply chains in terms of having upstream and downstream linkages, our method builds on the approach of Essington and Plagányi [18] which identifies key forage fish species in marine ecosystems. The Simpson Index [19] provides the underlying theoretical basis for both applications because it encapsulates both species richness (in this example, the number of different links present) and evenness (which is here replaced with the proportion of product that flows into each element in the supply chain). The network-type approach involves identifying the number of elements (or nodes), the number of links (or connections) and squaring the product “inflow” proportion to accord more weight to high throughflows that indicate important pathways in the system. Ranking individual scores allows scaling the importance of an element in the supply chain.

Globally, fish and fishery products constitute an important source of animal protein for humans and are among the most traded food commodities worldwide [20]. There is huge variability in the structure and types of supply chains, with many of these highly complex and increasingly globalised [20]. We demonstrate both the construction and interpretation of the SCI metric using an illustrative real-world application that focuses on the southern rock lobster *Jasus edwardsii* (SRL) fishery as well as the supply of four other Australian wild fishery seafood products to domestic and international markets (Table 1), namely the Torres Strait tropical rock lobster *Panulirus ornatus* (TRL), western rock lobster (WRL) *P. cygnus*, banana prawn *Penaeus merguiensis* component of the Northern Prawn fishery (NPF), and mixed fish from the Commonwealth Trawl Sector (CTS). An Australian farmed prawn and a Sydney rock oyster *Saccostrea glomerata* supply chain are also presented to illustrate the framework outside the wild fisheries sector. A sensitivity analysis is performed to explore behaviour of the SCI in response to changes in the structure of a supply chain. The results allow discussion of the potential stability and agility (ability for the supply chain to quickly re-adjust to changes in supply and demand conditions).

**Table 1:** Target seafood species for the different supply chain sectors considered in this study.

Industry Type	Sector Name	Species Common Name	Species Scientific Name
Wild (commercial) fishery (traps & pots)	Southern Rock Lobster Fishery (SRL)	Southern rock lobster	*Jasus edwardsii*
Wild fishery (diving)	Torres Strait Tropical Rock Lobster Fishery (TRL)	Tropical rock lobster	*Panulirus ornatus*
Wild fishery (traps & pots)	West Coast Rock Lobster Fishery (WRL)	Western rock lobster	*Panulirus cygnus*
Aquaculture (rock, stick and tray cultures)	New South Wales Oyster Aquaculture (NSWOA)	Sydney rock oyster	*Saccostrea glomerata*
Wild fishery (trawling)	Northern Prawn Fishery (NPF)	White Banana prawn; Red-legged Banana prawn	*Penaeus merguiensis; Penaeus indicus*
Wild fishery (trawling)	Southern and Eastern Scalefish and Shark Fishery (SESSF) - Commonwealth Trawl Sector (CTS)	Blue Grenadier; Tiger Flathead; Spotted Warehou; Orange Roughy; Pink Ling; Mirror Dory; School Whiting; Jackass Morwong	*Macruronus novaezelandiae; Platycephalus richardsoni; Seriolella punctata & S. brama; Hoplostethus atlanticus Genypterus blacodes; Zenopsis nebulosus; Sillago flindersi; Nemadactylus macropterus*
Aquaculture (ponds)	Australian Aquaculture Prawn Industry (AAPI)	Black Tiger Prawn	*Penaeus monodon; Marsupenaeus japonicus*

Overall, the simple approach to empirically-based supply chain research encapsulated in the SCI will be a useful addition to sectoral and industry-level logistics research. Most published supply chain evaluations have been qualitative in nature, utilising case-study approaches to understand organisation-specific responses [21]. The SCI, through the standardisation of supply chain components, the simple interpretation of the index, and the ability to easily identify key elements of supply chains allows future scenarios to be objectively evaluated. This is particularly relevant in the climate-change context where the socio-economic adaptations to future scenarios are often highly scenario specific, and responses may be beyond the control of individual organisations.

## Methods

### Developing models of supply chains

**Figure 1:**
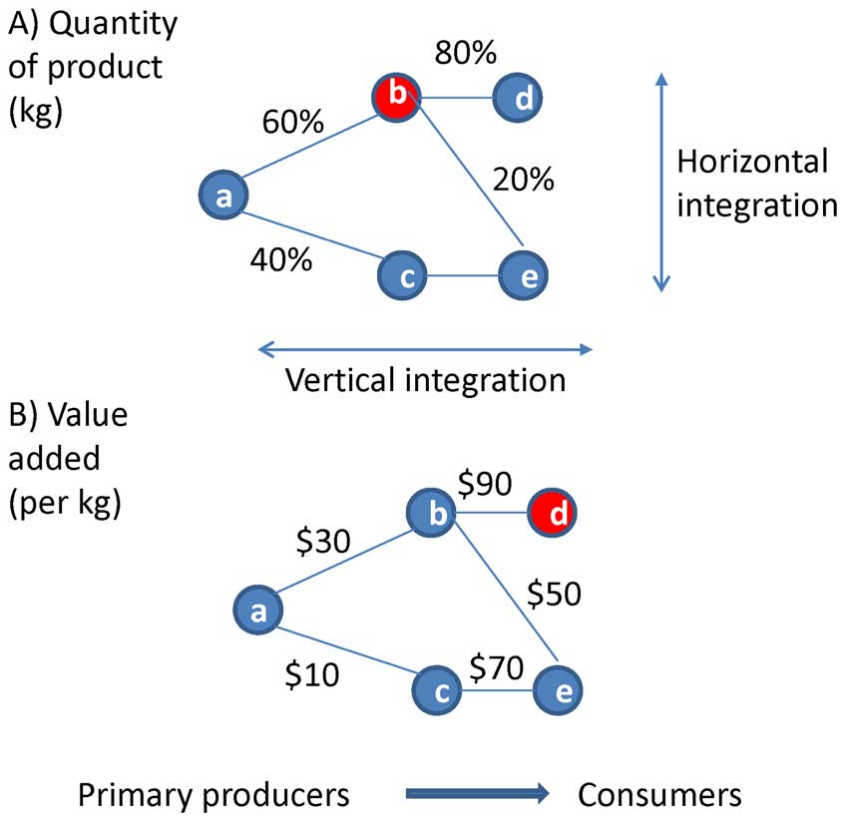
Schematic examples of supply chains. Links indicate (A) the proportion of the product (kg) that flows from one node to another, and (B) the value added along the chain per unit mass of product. Nodes represent the key stages in processing fish products, from the point where these are landed to the point at which they are consumed. The nodes highlighted in red are those identified as critical using the SCI described in the text. The figure also shows the difference between vertical and horizontal integration.

The first stage in the SCI analysis is to develop a model of the supply chain that captures the key processes and activities in the seafood sector, from the point at which goods (and services) are first produced to the point at which they are consumed (Figure 1). The models aim to represent supply chains in the seafood sectors in a simplified manner, enabling structured analysis, and acknowledging that behind these models are complex business structures and industry relationships.

**Figure 2:**
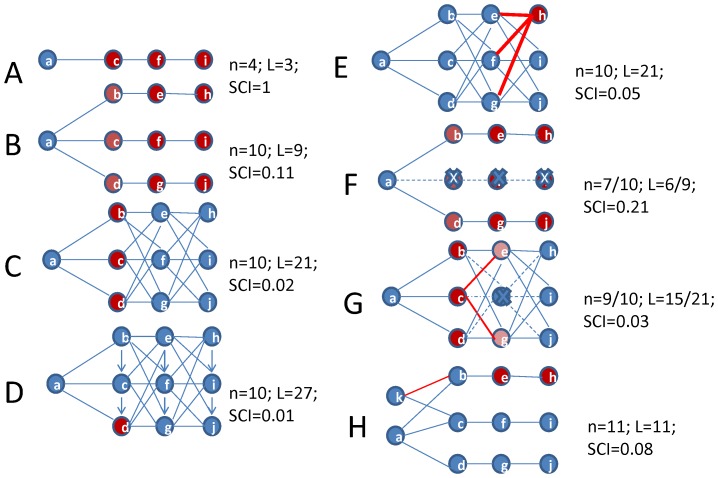
Schematic showing alternative hypothetical supply networks connecting a producer (a) to final consumers (far right). Supply chains/networks range from (A) linear through (B) parallel vertical paths, (C) cross-linked by progressively adding connections and (D) cross-linked with horizontal linkages. Sensitivities to these configurations include (E) unequal flows (red lines), (F), removing vertical layer of nodes (c, f and i) (G) removing node (f) and (H) adding an additional producer (k). The links are all assumed of equal magnitude except in (D) depicted by arrows represent 10% of the product flowing in the direction of the arrows, and the red lines indicate a relatively larger flow of product. Each chain has *n* nodes, *L* links and the standardized Supply Chain Index (*SCI*) is shown alongside. Critical elements are identified as those with the highest individual SCI scores and are highlighted in red.

The development of a supply chain model parallels the development of a trophic network representation, where species and groups of species, and the connections between them, are identified as a *n* × *n* matrix, where *n* is the number of elements (or nodes), and there are *L* non-zero elements, where *L* is the number of links (or connections) in the network. The graphical model of a supply network presents components which are the key economic agents involved in the process, and the connections between them, in terms of the quantities and/or values of fish processed (see e.g. Figure 1). Supply chains will differ predominantly in terms of the number of elements *n* and links *L*, the connectedness (or complexity) of the system (Figure 2), as well as the functional and other topological properties of food-webs -e.g. path lengths, clustering coefficients, degree distributions etc. [22], [23].

The level of detail captured by a supply chain model will be driven by a combination of the understanding and information available about the systems considered, as well as the main questions driving the analysis (in this application, the identification of critical elements that might be most at risk from the impacts of climate change). The analysis may also differ depending on the resolution (number of elements) chosen to describe the different components of a supply chain. If several supply chain models are to be compared, it is important that their resolution be comparable and that metrics used for comparative purposes are reasonably robust to slight differences in the degree of aggregation of individual elements comprising a supply chain. Analogously, in the ecological literature, several studies have focused on selecting the most appropriate level of resolution as well as highlighting the sensitivity to alternative configurations of conclusions drawn [24], [25]. Hence in both ecological models and supply chain models, consideration needs to be given as to the degree to which individual state variables (such as species, functional groups, processes or companies) are aggregated into a single state variable, noting the need for adequate resolution describing key levels (for example, trophic levels in a food-web and proposed structured steps linking producers to consumers in supply chains).

### Metrics to characterise a supply chain

The simplest metrics to characterize a supply chain network are the number of elements *n* and links *L*. In addition, two fairly conventional metrics are computed as measures of the connectance of a supply chain, the links per node ratio
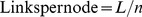
(1)


and, analogous to food-webs, connectance, which measures the interaction richness as follows:
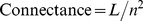
(2)


Next we derive a new index (which we term the Supply Chain Index) that incorporates weighting of links as follows. Given a model of a supply chain, for each component along the chain, we compute first *s_ji_* which represents the proportion of total product that receiver *j* receives from supplier *i* relative to all product flowing into that element *j*, such that for receiver *j*, 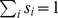. These proportions could, for example, be expressed in terms of quantities received from this supplier, as a proportion of all quantities received. This will be used to measure the “spread” of product flows into a receiving element.

A second variable, *p_j_*, measures the proportion of the total product in the supply chain that flows into receiver *j*, such that the product of the two measures represents both connectance and importance or magnitude of flow. Hence, for example, if there is a single link from a processor to an internal exporter (*s_ji_*  = 1) the product of 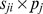 will give a higher ranking to the exporter that handles 50% of the product than to one who handles only 5% of the total product. Moreover, squaring the term *p_j_* in equation 3 assigns more weight to important elements, and also effectively reduces sensitivity to the level of aggregation of the supply chain elements.

The proposed Supply Chain Index (SCI) for each element *j* is thus:
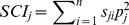
(3)


with critical elements then identified as those with the highest SCI score(s).

The overall Supply Chain Index Total (SCIT) for the supply chain as a whole is obtained by summing over individual scores:
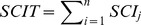
(4)


The index is then standardised by dividing SCIT by the number of links, *L,* thereby allowing comparisons across supply chains., i.e. 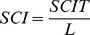
(5)


The minimum value for each SCI_j_ is zero (in which case the element is not receiving any product) and the maximum theoretical value is one which would imply a single receiver of all products (i.e. 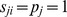). Similarly the maximum theoretical value of the SCIT is *L*, and hence dividing by *L* scales the maximum value to one (i.e. in the case of a purely linear system).

The index uses as a starting point products flowing outwards from producers. Producers themselves will be accorded a score of zero, but will be able to identify key components of the rest of the supply chain based on the highest scores. The illustrative analyses here are all presented in terms of quantity or volume of product, but the same analysis could be applied if data were available on the value added at different stages as a product flows through the chain. Components of the system that generate higher value added could then be considered critical elements instead and incentives for change targeted towards these.

### Worked examples

To illustrate the calculation and interpretation of the SCI, a simple example is presented below, with the first model (Figure 1A) showing the flows along a hypothetical supply chain in terms of the quantity (or volume) of product, and the second model (Figure 1B) superimposing monetary units of value added. Step 1 involves constructing a matrix with elements, *s_ji_* representing the proportion of product that receiver *j* (column headings) receives from supplier *i* (row headings) relative to all product flowing into that element *j* (columns must therefore sum to one) (Table 2). The matrix has an order of *n* × *n*, diagonal elements are zero and the number of links, *L*, corresponds to the number of non-zero elements (five in this example). For example, node (b) receives 100% of its product from node (a) whereas node (e) receives 40% of the total product from node (a) (via node (c)) and 12% (20% of 60%) via node (b), so that the proportion that flows into node (e) from node (b) is 0.23 (computed from 12/52).

**Table 2:** Example of Step 1 to compute the SCI: calculation of the proportion of product that flows into a node from different nodes, for the example of a chain presented in Figure 1A.

	a	b	c	d	e
a	0	1	1	0	0
b		0	0	1	0.23
c			0	0	0.77
d				0	0
e					0

The second matrix shown in Step 2 (Table 3) captures the relative quantity of product that flows to each receiving element. For example, node (c) receives 40% of the total mass of product originating from node (a). Step 3 involves multiplying corresponding elements in the two matrices after squaring each element in matrix 2 (Table 4). Summing each column in the resultant matrix yields the SCI_j_ scores for each node in the supply chain, and key elements are then identified as those with the highest SCI_j_ score/s. The key element (highlighted in red in Figure 1A) is identified as node (b) both because of its connections and because it handles a large volume of product. Finally the SCIT for the supply chain is computed as the sum of the SCI_j_ scores, and the standardised SCI value calculated as in equation (5), where the number of links in our example is five (Table 4). The values of the SCI will range from one (for a strictly linear chain) to a minimum of approximately 0.01 (for a highly connected chain).

**Table 3:** Example of Step 2 to compute the SCI: calculation of relative proportion of total product that flows to a node.

	a	b	c	d	e
a	0	0.6	0.4	0	0
b		0	0	0.48	0.12
c			0	0	0.4
d				0	0
e					0

**Table 4:** Example of Step 3 to compute the SCI: Product of first matrix and square of the second.

	a	b	c	d	e
A	0	0.36	0.16	0	0
B		0	0	0.23	0.00
C			0	0	0.12
D				0	0
E					0
SCI (element)	0.00	**0.36**	0.16	0.23	0.13
	**SCIT**	0.88	**SCI(std)**	0.18	

If data are available on value added, additional analyses as described in Tables 5–7 could also be performed. Value added is defined as the amount by which the value of a product is increased at each stage of its production, exclusive of initial costs. First a value added matrix is constructed as shown in Table 5 based on the example in Figure 1B. It should be noted that the matrices in Tables 5–7 cannot be interpreted as in Tables 2–4 because, for example: despite a value of $$30 being reported at the intersection of element (b) and row (a) this denotes the added-value created by the primary producer, node (a) (Figure1B), selling their product to node (b). The additional value is created by, and accrues to, node (a) but only as it is sold to (b), and the resulting matrices should be interpreted with this in mind. The quantities of product that pass through different value-adding nodes vary according to the values given in the Step 2 matrix (Table 3) above. Hence multiplying corresponding elements of the matrices defined in Table 3 and Table 5 gives the relative value added per mass or quantity of product (Table 6). The total value added by the i^th^ node is provided by summing across each row in the matrix of Table 6. Thus, Table 6 illustrates that for every 100 kg of product that passes through this system $$9920 of value-added is realized; $$2200 by node (a), $$4920 by node (b), and $$2800 by node (c). The value-added of alternative paths may also be calculated, e.g. (a) → (c) → (e)  =  $$5000 (or, (a) → (b) → (d) & (e)  = $$7120).

**Table 5:** Example of method to compute the SCI^V^: value added per kilogram flowing into different nodes, for the example of a chain presented in Figure 1B.

	a	b	c	d	e
a	0	30	10	0	0
b		0	0	90	50
c			0	0	70
d				0	0
e					0

**Table 6:** Example of second step in method to compute the SCI^V^: product of proportion and value added per kilogram.

	a	b	c	d	e	value-added by *i*
a	0	18	4	0	0	22
b		0	0	43.2	6	49.2
c			0	0	28	28
d				0	0	
e					0	
						99.2

**Table 7:** Example of third step in method to compute the SCI^V^: product of Table 2 matrix and square of Table 6 matrix.

	a	b	c	d	e
a	0	324	16	0	0
b		0	0	1866.2	8.3
c			0	0	603.7
d				0	0
e					0
SCI (node)	0.0	324.0	16.0	1866.2	612.0
	**SCIT^v^**	2818.2	**SCI^v^(std)**	0.05	

As before, the final step (Table 7) involves multiplying corresponding elements in the Table 2 and Table 6 matrices after squaring each element in the Table 6 matrix. Summing each column in the resultant matrix yields the individual value-added SCI^v^ scores for each node in the supply chain, and nodes with the highest SCI^v^ score/s are key nodes in terms of value adding from the upstream node (Table 7). Hence the relationship between nodes (b) and (d) is identified as a key element from an economic perspective. The total SCIT^v^ index for the supply chain is again computed as the sum of the individual scores, but the standardised value is computed as the total divided by the square of the sum of each column in Table 5.

### Illustrative applications of the supply chain index (SCI)

To illustrate how the SCI works, as well as validate that it performs as expected, eight hypothetical supply chain models and sensitivities were used (Figure 2):

- A simple linearly integrated supply chain that is efficient on the one hand but provides limited alternatives if any one linkage breaks;
- Parallel pathways to three different receivers providing the producer (e.g. fisher) with alternatives and some form of competition for product but after the first column the next nodes in the chain are linear;
- More cross-links providing opportunities and competition for product along the supply chain;
- Increased diversification of product or receivers specialising in a certain ‘product type’ (e.g. second rate product) thus increasing the relative value added component. In this example it is assumed that 10% of the product in each row flows directly to the corresponding node in the row below.
- Unequal flows in the supply chain (in contrast to the above examples which assume equal flows). In this hypothetical supply chain, the top node (h) in the last column is assumed the most favoured in that column (as indicated by thicker red lines in Figure 2E), with half of the product from each of the downstream suppliers assumed to flow to this node. This may be due to some advantage (e.g. paying a higher price) and thus there may be some cost or penalty associated with shifting to node i or j in the last column.
- As in (B) but with the removal of a central node (f), which essentially removes a vertical layer of nodes and links as indicated.
- As in (C) but with the removal of one node (f) and associated links.
- As in (B) but with the addition of another primary producer (with node k producing 50% as much as node a), and preferentially supplying (red line in Figure 2H) the top node in the second column and one other node.

The number of nodes *n*, links *L* and the SCI are computed for each case. Critical elements are identified based on the highest SCI_j_ scores and are highlighted in red. In all the cases considered, a key assumption is that the components of the supply networks operate as separate businesses in the economy, hence links between them involve contracts between separate business entities. Where some vertical integration exists, e.g. through shared ownership of businesses operating at various levels in the network, it might be necessary to consider these as single entities.

### Application to real-world Australian supply chains

The index has been applied to seven Australian supply chain examples: southern rock lobster *Jasus edwardsii* (SRL), Torres Strait tropical rock lobster *Panulirus ornatus* (TRL), western rock lobster *P. cygnus* (WRL), New South Wales Oyster Aquaculture (NSWOA) of Sydney rock oysters *Saccostrea glomerata*, banana prawn *Penaeus merguiensis* component of the Northern Prawn fishery (NPF), mixed fish from the Commonwealth Trawl Sector (CTS) and prawns from the Australian Aquaculture Prawn Industry (AAPI) supply chain. The supply chains for each of these seafood industries were constructed to include a common set of levels or components: fishers, interim storage, fish receivers, interim transport, interim storage, primary wholesale, secondary wholesale, domestic market and export destinations, and consumers. Information available on the quantities of product flowing between agents along the supply chain was used to characterise the links between elements or nodes. Mapping these supply chain flows served as a basis for developing the supply chain models for each industry.

The SCI_j_ scores for all elements in each of the supply chains are calculated and ranked and critical elements identified (within and between supply chains). The impact of potential changes to each supply chain is investigated by calculating the effect on the score of altering values of *p* and *s.* The SCIT (Equation 5), as well as the distribution of the SCI_j_ scores for each chain provides information on the characteristics of the chain.

A pie graph shows the distribution of these scores at a glance across the different components of each supply chain, with the size of the pie slice depicting the importance of each element's score for elements comprising 1% or more of the total summed score. From highest to lowest scores, the colour coding used is red (>20%)-orange-green-blue-purple. Additional highlights have been added on the supply chain schematics to the red and orange boxes, to emphasize where the critical elements are and how they are distributed.

### Interpreting the supply chain metrics

The SCI method yields a consistent and objective set of metrics for evaluating and comparing supply chains, but the interpretation of these depends on a number of factors. The SCI_j_ scores identify key elements but do not inform whether it is better to add resources to safeguard these key elements, or introduce new linkages to spread the risk of the chain collapsing in response to a shock to the system.

The SCI itself is not intended as an overall measure of the optimality or otherwise of a supply chain as clearly a number of factors collectively determine what is better or worse in terms of supply chain structure. Conventionally, in a supply chain context, the more suppliers or buyers there are, the more likely it is to be transaction-based, and therefore, less meaningful business relationships ensue so that a streamlined supply chain with fewer linkages or connections may be preferred. On the other hand, a diffused or fragmented chain involving multiple steps may be inefficient in some contexts, but have advantages in other situations. Models of optimal supply chain design generally find that an optimal network has fewer elements than a “naturally” evolving network when costs of moving product from one element to the next are linear, but with non-linear costs a “naturally” evolving network with many linkages between elements may be efficient [26].

In the context of this paper, we consider a decrease (lower score) in the SCI an improvement in terms of the resilience and ability to adapt to shocks and changes. In general a lower score indicates greater connectivity, as well as greater resilience to external shocks such as changes in the spatial and temporal distribution of fishery production in response to changing climate. A high score may reflect that a supply chain depends critically on a few elements only. To interpret further what the implications are in terms of the chain's agility and hence ability to respond and adapt depends on the economics of the specific case study and individuals and organisations involved. For example, a diffused supply chain might be a sign of a market that is competitive, mature and complex, thus the need for a wide range of actors playing different parts, and encompassing high capacity to shift and adapt to shocks to the system. Such an interpretation of a diffuse supply chain implies a lack of economies of scale which, in other contexts, may be associated with lower aggregate costs of adapting than would be the case for a larger number of smaller elements.

To some extent what is ‘good’ or ‘bad’ depends on the type of adaptation option that may be put in place – for example, if there are only one or two really critical elements, it might be much easier to adapt the system, even if it was more vulnerable initially. Conversely, if product is distributed via ten important elements then each element may involve a different adaptation approach or option (each with different assessment, monitoring etc.) which may make it more difficult to introduce/implement bigger or more meaningful/effective changes.

## Results

### Schematic illustration of the SCI

The SCI calculation uses the square of the *p_j_*. Hence, if a large volume of product flows through a single element compared with a scenario in which half this amount flows through each of two elements, then the *p_j_* ^2^ contribution to the SCIT for the first casewill be twice that of the latter, i.e. higher scores suggest greater dependence on fewer elements.

As expected, the progressive addition of more linkages (cases A-D in Figure 2) results in a (non-linear) decrease in the SCI, with lower scores indicating that a supply chain is more connected. The values of the SCI range from one (for a strictly linear chain) to 0.01 (for a highly connected chain) (Figure 2 A–D). The simple links per node metric also captures the increase in connectance, with the values for cases (A)–(D) increasing from 0.75 through 0.9, 2.1 and 2.7, but these values are arguably harder to interpret than the standardised SCI values. The connectance metric captures the increase in connectance as one moves from case (B) to (D) in Figure 2, with scores increasing from 0.09 to 0.21 and 0.27, but the high score of 0.19 for case (A) is not consistent with the pattern and is less meaningful in this instance.

For cases (A) and (B) there was nothing to distinguish between the supply chain nodes, and hence these are equally identified as critical elements. For case (C) the first column nodes are identified as critical nodes whereas in case (D) the first bottom row node emerges as the key element (as expected) because more product flows through this node due to the vertical downward linkages assumed to flow towards this node.

Simulating the effect of unequal flows in the supply chain (Figure 2, case E) resulted in the most favoured node being (correctly) identified as a key element because a large proportion of the product from several upstream nodes flows to that node. Although the number of nodes and links in case (E) was the same as for case (C) the SCI increased from 0.02 to 0.05 in response to the flows becoming unequal. This is consistent with the expectation that a lower SCI represents greater connectivity, but also greater stability because of the ability to disperse shocks and impacts to the system. The simpler links per node and connectance metrics remain the same in both cases and hence are less informative than the SCI.

The next sensitivity involved removing node (f) from each of cases (A–C), although in the trivial case (A) the chain collapses (because there is no longer a viable linkage between the producers and consumers). In case (B), removing central node (f) also results in node (i) becoming redundant (under the simple assumption here that no replacement links are initiated with other nodes), and similarly node (c) has no alternative connections upstream so becomes redundant also (Figure 2, case F). This results in an almost doubling of the SCI reflecting a less connected network. In contrast, removing node (f) from case (C) does not have similar repercussions for nodes (c) and (i) because they have alternative downstream and upstream linkages and it is assumed here that a larger volume of product is simply redirected along these existing pathways (case G). Overall one node is lost together with six links, but in this case there is only a small increase in the SCI (from 0.02 to 0.03) because the network is still relatively highly connected. Hence supply chain (G) is arguably more stable or resilient to change than supply chain (F), and hence in general a lower SCI score reflects a more structurally stable or resilient supply chain. Note though that other considerations may be important also, such as the economic efficiency or overall carbon footprint [27], [28] and other ecological as well as strategic business considerations of a supply chain. The index does not inform on these aspects such that a lower SCI score does not necessarily indicate an optimal supply chain configuration from an overall socio-ecological perspective.

The final sensitivity (Figure 2, case H) explored the impact on case (B) of adding one more primary producer (increasing connections at the producer end of the chain) and changing flows to be unequal to preferentially supply (red line) node (b). This changes the identification of the key elements to nodes (e) and (h) given the majority of the product now flows through this pathway. The increase (from 9 to 11) in the number of links in this model results (as expected) in a decrease in the SCI on the one hand, which is offset slightly by the skewed distribution of flows in the network (approximately 50% of the product is channeled to consumer (h) in this example) so that overall there is a slight decrease (from 0.11 to 0.08) in the SCI (Figure 1H). In general, the addition of a producer (node earlier in the chain) will have a bigger impact on improving (i.e. decreasing) the SCI than adding a consumer (less connected node at the top end of the chain). For example, if instead of adding a producer to case (B), an additional final consumer is added so that product from node (e) is now shared between node (h) and the new final consumer (not illustrated here), the SCI will decrease slightly from 0.11 to 0.09 (i.e. not as much as for case (G)).

### Southern rock lobster (SRL) supply chain case study

**Figure 3:**
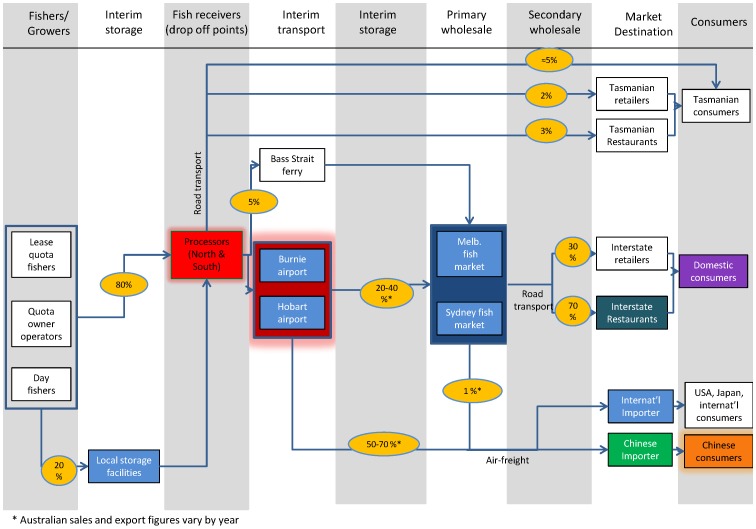
SRL supply chain model configuration. Colour coding highlights key elements in the SRL supply chain identified using the SCI, with the relative distribution of these summarised in the pie diagram in Figure 4.

Southern rock lobsters (SRL) in Tasmania are fished by a combination of lease quota fishers, quota owner fishers and temporary day fishers. Most of the product from fishers is sent to processors located in Tasmania, who then send the majority of the product to Australian mainland markets (primarily Sydney and Melbourne) and international destinations (primarily mainland China) (Figure 3). A number of sources of vulnerability of SRL to climate change drivers have been identified [29]. These include impacts from large-scale declines in puerulus recruitment correlated with changes in large-scale oceanographic features [30], changes in local environment conditions affecting growth of lobsters and increased overlap with southwards migrating species such as octopus which increase predation pressure. Collectively these changes will affect the production and spatial distribution of lobsters, which in turn impacts on supply to processors.

Identifying key elements in the chain may assist in informing adaptation strategies to reduce exposure to anticipated future risks posed by climate change and other risks such as shifting market forces. The key elements identified through computation of the SCI_j_ for the SRL supply chain are respectively, the airports (Hobart and Burnie), the processors, and Chinese consumers (Figure 3; 4A). These elements are key because of the volume of product that flows downstream from upstream suppliers. Ensuring the resilience of key elements in a chain may be particularly important in maintaining the longer term stability of a supply chain. On the other hand, an alternative way to strengthen the chain is to deflect or spread the criticality to other parts of the chain, such that the SCI_j_ scores can help highlight the need to reduce risk associated with having a critical element. Hence for SRL, this analysis highlights that it might be fruitful to explore options for alternative Australian transport hubs, including Tasmania's major airport (Hobart airport) or alternative routes through other major cities, as a means of increasing the key “airport” nodes flexibility to shift and adapt. Moreover, emphasis could be placed on supporting and building the resilience of other key elements such as the processors and Chinese consumers. For example, focusing effort on firmly establishing Chinese trade agreements may be one critical area providing scope for growth and building stability in the SRL supply chain.

Closer to the producer end of the chain, the processors are highlighted as important elements and hence the resilience of the chain can be strengthened by focusing interventions on building the stability of this component. For example, contingency plans could be put in place to diversify product types that are more versatile in terms of ‘storability’ (for example, converting fresh product to frozen tails) thus making it possible to even out seasonal distribution of their product in anticipation of climate-driven environmental impacts. An example of a recent climate-related shock to the system is the closure of the SRL and other south-east Tasmanian fisheries in 2013 in response to a toxic algal bloom associated with warming water and enhanced transport by ocean currents (see [31]). As a consequence of the fisheries closure the processors experienced reduced product throughput with resultant financial implications. This supports the finding of the model that the processors may be vulnerable to climate risks if they have not developed strategies to build resilience to periods of reduced supply. For example, they could change from predominantly live product to more stored product (i.e. cooked or tailed) thus making it possible to even out seasonal distribution of product in anticipation of climate-driven environmental impacts. Other links such as between the storage facility and processor may also be threatened if dips in the supply connecting these nodes become more frequent or prolonged. Firstly there may be a risk that another Tasmanian fish product (i.e. a finfish species or scallops) is channeled through this processor node instead and takes up excess capacity. Secondly there is a risk to the entire chain if another supplier of a different Australian lobster species (TRL or WRL) replaces the SRL suppliers at the consumer end of the chain. This second risk is all the more important if the product flow through the chain is interrupted frequently enough (e.g. by climate change shocks), especially as climate change impacts are expected to vary substantially between different Australian states, and the three major export species are considered to be substitutes [32]. Greater collaboration between producers of different Australian species supplying the same markets may be one strategy to increase resilience of these supply chains.

The SCI for SRL is 0.09, which is about mid-range compared with other supply chains explored in this study, as well as with alternative sensitivity scenarios for the SRL chain itself (Table 8).

**Table 8:** Illustrative sensitivity scenarios applied to the SRL case study.

Sensitivity name	Description
Base Case	Current model
Sensitivity 1	Key element (airport): reduce the dependence on Hobart airport by assuming that half the product is transported instead via the Bass Strait ferry;
Sensitivity 2	Chinese Consumers: reduce the amount of product flowing to the international Chinese market, and redirect it to the local Australian mainland consumers instead;
Sensitivity 3	Domestic Consumers: as in C), but further remove Tasmanian consumers link such that almost all product flows to Australian mainland consumers.

### Sensitivity scenarios

The sensitivity of the SCI in response to changes in the structure of the SRL supply chain was explored using scenario analysis (Table 8). The first scenario investigates reducing dependency on the critical element as identified above. Two ‘demand-driven’ scenarios are also developed both of which involve an increase in the importance of the domestic Australian market which might occur if for some reason (collapse of trade agreements, decrease in perceived quality of Australian lobster, health scare due to algal blooms), there is a reduction in the outlets for SRL on the Chinese market. An implicit assumption in the sensitivity analysis is that there are no capacity constraints at any point in the supply chain, such that product can be redirected as indicated, noting that this is overly simplified but intended as an illustration of the method.

**Figure 4:**
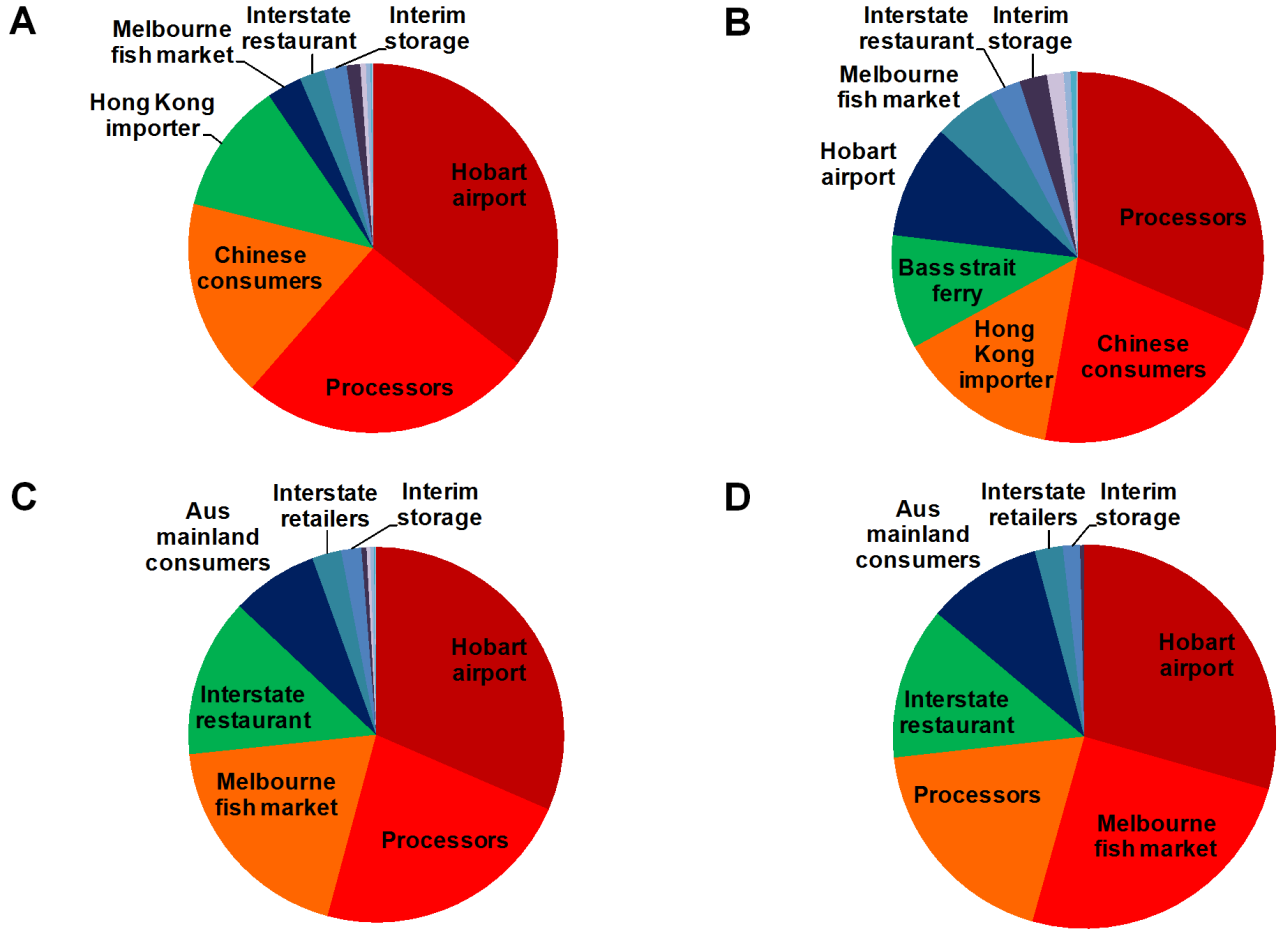
Sensitivity analysis to compare relative *SCIj* scores for components. Current model (A) is compared with three sensitivity scenarios (B) – (D) (see text for detailed descriptions) using the SRL supply chain as an example.

Under scenario (B) described in Table 8, the SCIT decreases from 2.02 to 1.65 and SCI from 0.09 to 0.075, indicating an improvement in the stability and robustness of the chain as the critical dependence on the airport is relieved to some extent through the (hypothetical) introduction of an additional important transport element. The SCI_j_ of the airport node is reduced so that under this sensitivity the processors and Chinese consumers become the most critical of the elements (Figure 4B). This scenario demonstrates the improvement in resilience which can result from lessening the dependence on a single key element and strengthening or adding alternative complementary pathways and connections.

Scenarios (C) and (D) in Table 8 explore the effect of narrowing the current distribution of product from several final consumers to progressively fewer, first by substantially decreasing the flow to international markets and secondly by completely removing the Tasmanian consumer pathway. As expected, the SCI metric worsens from the base-case value of 0.09 to 0.10 under (C) and to 0.13 under (D). The Chinese consumers lose their ranking as one of the key elements and the Melbourne fish market becomes relatively more important instead (Figure 4C). Note that the importance of these effects is partially related to the fact that the indices were calculated based on quantities exchanged between agents, and would only hold if the expected value of products re-routed towards the Australian domestic market remained equivalent to what it is on the external, largely Chinese market. If this was not the case (i.e. returns from lobsters sold on the Australian market are lower than the Chinese market), then the expected effects of the change in the supply chain would not be as great, if measured in value terms.

In the first instance, this scenario highlights that reducing connectivity and linkages in a supply chain increases its dependence on single agents, which may have important consequences in terms of overall stability and agility. Where the unit costs of adaptation are independent of the scale of the elements, supply chains with less connectivity and linkages may be less stable and agile. In cases where economies of scale affect the costs of adaptation, supply chains with fewer, larger elements may be more stable and agile. Moreover, it is arguable that in an export market (and depending on a number of factors) having fewer larger players could put the industry in a stronger position with respect to controlling profit margins. In this simplistic example, the removal of elements, and hence reduction in total path distance, does not compensate in the SCI score for the negative effect of reducing connectivity (because the SCI score accords higher weight to connectivity).

### Comparisons across multiple fisheries examples

The supply chain models for the additional examples considered (Table 9), are shown in Figures S1, S2, S3, S4, S5, S6 in File S1, and these examples are not discussed in detail in this manuscript (but see Supporting Information Material File S1). Rather the focus is on an illustrative use of the SCI as a standardised metric for making comparisons across supply chains (Table 9, Figures S7, S8 in File S1).

**Table 9:** Summary of supply metrics, including simple measures (links per node and connectance) and the standardized Supply Chain Index (*SCI*), plus top three key elements identified based on individual *SCI_j_* scores, for the seven case studies as shown (see File S1) for supply chain model configurations).

Supply chain	No. nodes *n*	No. links *L*	Links per node *L/n*	Connectance *L/n^2^*	Supply Chain Index Total *SCIT*	*SCI* (standardised)	Key Element 1	Key Element 2	Key Element 3
SRL	17	22	1.29	0.08	2.02	0.092	Hobart airport	Processors	Chinese consumers
TRL	15	16	1.07	0.07	1.35	0.084	Chinese importer	Chinese consumers	US importer
WRL	22	33	1.50	0.07	1.59	0.048	Chinese consumers	Chinese importer	Processors (Geraldton)
NSWOA	13	19	1.46	0.11	2.58	0.140	On-farm storage	Ute/truck	Sydney/Brisbane
NPF	15	28	1.87	0.12	0.64	0.023	Supermarkets	Domestic consumers	Mother ship
CTS	14	18	1.29	0.09	1.99	0.110	Co-op business	Melb/Sydney markets	Retailers (fresh)
AAPI	10	16	1.60	0.16	1.12	0.069	Domestic consumers	Chain/indep. retailers	Primary wholesaler

**Figure 5:**
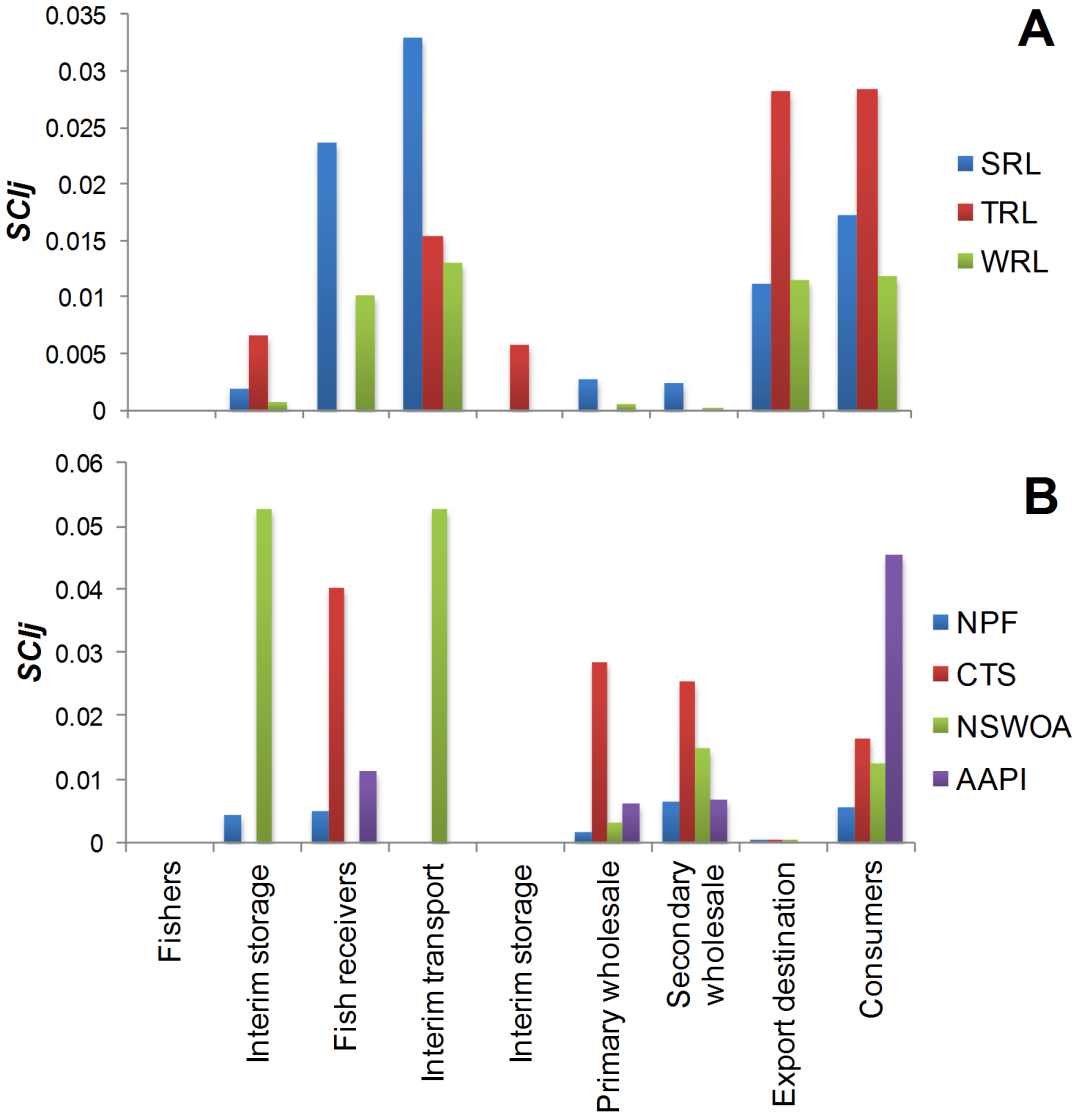
Plots of the standardised *SCI_j_* metrics aggregated over different stages *j* of each supply chain. The plot compares the distribution of key stages in each of the wild seafood and aquaculture supply chain case studies. See Table 1 for summary of acronyms used.

The WRL supply chain has the largest number of nodes and links, but the highest ratio of links to nodes is seen for the NPF supply chain, suggesting it is highly connected. The most direct (lowest links:nodes ratio) supply chain is the TRL (Table 9). The highest and lowest SCI scores were for the NSWOA and NPF chains respectively. The top three key elements in each supply chain, as identified using the SCI_j_, differed across all the case studies, with the most common element being consumers (whether domestic or international). Across these seven supply chains, four had key elements at the downstream (consumer) end (TRL, WRL, NPF, AAPI) and three at the upstream (transportation and storage) end (SRL, NSWOA, CTS) (Figure 5).

## Discussion

Food-web matrices constitute important foundations to almost all fisheries ecosystem modeling, and considerable effort has focused on deriving quantitative descriptors to facilitate understanding of the structure and function of the underlying ecosystems and their components [24], [33], [34], [35]. Supply chains can also be viewed as networks and matrices [2] and describing their structure can be constructed in an analogous manner. In this paper we draw on an approach developed to identify key species in ecological networks [18] for use in identifying key elements in seafood supply chains, with potential application to broader examples. Analogous to measuring the magnitude and connectedness of trophic interactions, the SCI measures the connectedness of supply chains and magnitude of product flowing downstream. The application to a supply chain is even more ‘direct’ in the sense that in a trophic network energy is lost in each transfer up the chain but there is no such loss as product travels through the supply chain. In the same way that different economic value can be assigned to pathways in a trophic food-web, for example to quantify the economic trade-offs in fishing forage fish versus leaving them as prey for more valuable higher level predators (e.g. [36]), economic information can be assigned to links in a supply chain model for use in analysing the efficiency or economic optimality of different structures. Additional units which could be explored in future research include revenue and societal cost (e.g. [37]) and the energy use and carbon footprint associated with alternative network pathways (e.g. [28]). In addition, there are also analogies between the food-web and supply network metrics outlined here, and the many similar statistics employed in social network analyses [38], [39], [40]. Constructing models of supply chains in a consistent and structured manner and calculating a standardised SCI index facilitates examination of a supply chain from a range of perspectives from within the chain, and allows inter chain comparisons.

A simple hypothetical example was used to illustrate the (non-linear) decrease in the SCI in response to the progressive addition of more linkages in a supply chain, with lower scores indicating that a supply chain is more connected. The values of the standardised SCI range from one (for a strictly linear chain) to 0.01 (for a highly connected chain), and are therefore comparable across different examples (Figure 1). In general, the addition of a producer (node earlier in the chain) was found to have a bigger impact on improving (i.e. decreasing) the SCI than adding a consumer (less connected node at the top end of the chain).

Lower overall SCI scores suggest a supply chain that relies on more diffuse links between its agents. Where adaptation requires an ability to quickly shift the flows traded from one path to another, and the costs of doing so are not scale dependent, then it might be expected that the supply chain would be more resilient to externally imposed changes. On the other hand, supply chains with higher overall SCI scores, implying a stronger dependence on single agents concentrating an important part of material flows may be more effective at adapting if the adaptation costs are scale dependent.

Supply chains are challenged by constant change in product availability, domestic and international markets and other shocks to the system. From the point of view of Australian fisheries and aquaculture producers, sustainability is largely determined by the ability of these supply chains to adapt such that the outlets for seafood products are maintained in the future. An increasingly important challenge to supply chains involves building resilience to changing climate. Recently Levermann [41] highlights the need to make supply chains climate-smart in part by analysing their connectivity and identifying which links or nodes may be fragile, and hence where best to focus attention. Our approach provides one method for characterising supply chains, and identifying the key agents in these chains which determine the likely response of a supply chain to external shocks. Our illustrative simulations using the SRL example and a recent climate-change related shock in the form of an algal bloom that closed the fishery, highlight the changes which can result from lessening the dependence on a single key element and strengthening or adding alternative complementary pathways and connections. The approach can also be used as a tool for supply chain design and redesign strategies, especially where a specific risk is encountered at particular supply chain stages.

Moreover, scenario analysis indicated that reducing connectivity and linkages in a supply chain may decrease its stability and agility (but note also the caveats outlined in the Methods). In the SRL example the removal of elements, and hence reduction in total path distance, has less of an effect on the SCI score than the negative effect of reducing connectivity. This is because the SCI score accords higher weight to connectivity than to other measures such as the number of elements. The fact that the entire supply chain did not collapse when first one and then two pathways were reduced and removed, suggested that the SRL supply chain is reasonably robust, in the sense of being able “to resist change and preserve connectivity after nodal removal” [42]. In these illustrative sensitivity scenarios, it was assumed that Australian mainland consumers would be able to absorb additional product (and pay a reasonable price), but in reality the resilience of this supply chain strongly depends on the extent to which this assumption holds. While transport costs would be significantly reduced, increased supplies to the domestic market may also result in a substantial decrease in prices received. The application of the SCI will therefore be most useful in combination with market demand [14] and supply analysis and supplemented by qualitative assessment of each supply chain phase. Recently the modeling software Ecopath with Ecosim has added a capability to keep track of the flow (amounts, revenue and costs) of fish products from the point of capture to the end consumer [43]. Such analyses are extremely useful to evaluate the trade-offs between different fisheries, cross-linkages and important components [43], but were not designed to quantify connectivity and resilience attributes.

Previous analyses of the SRL supply chain, as well as the other examples presented here, have been largely qualitative in nature, such as an evaluation of the economic resilience of the SRL fishery by van Putten et al. [44]. Their in-depth analysis of the linked biophysical and human systems identified the following three areas of potential low economic resilience to climate change: diversification, information flow and sectoral climate change plan. The strong reliance on a single market and need to diversify to protect against closure of a key market corroborates well the findings from this study that the Chinese market is a key critical area. By quantifying and analysing the structure of the SRL chain using the SCI method, a contribution is also made towards improving the latter two attributes, namely information flow (flow of the product, supply chain transparency [45]) and planning for climate change impacts. Similarly, an earlier analysis of the TRL supply chain was semi-quantitative in terms of mapping the flow of product and hence permitting computation of the value added [46], but could only provide qualitative descriptions of critical elements in the chain.

In the Results section we provide some illustrative interpretations only, as the basis of the metric is throughput and dependence on individual elements of the chain, and the aim here is to preliminarily assess the ability to respond to shocks and changes to the system. Without superimposing additional economic information, it is not possible to comment on the overall efficiency of a chain. There are some potential conflicts between the possible need to maintain alternative diffuse connections in a supply chain and reducing the number of pathways to perhaps optimise the efficiency of a chain. By splitting pathways, economies of scale may not be reached, increasing costs significantly while the need to propup new businesses (through subsidies) may generate false economies. Hence our analysis provides insights into the structure of supply chains and key elements, but whether the most suitable method for strengthening and increasing the resilience of a chain involves strengthening individual elements or adding new pathways will also depend on other factors such as economies of scale.

Our finding that in some contexts a lower SCI may be associated with increased resilience is equivalent to the finding that robustness increases with connectance in ecological networks [22]. Both anthropogenic influences and climate change threaten biodiversity in ecological systems, driving the need for research to understand the role of species richness in contributing to the stability and functioning of ecosystems [22]. Moreover, Dunne et al. [22] show that the loss of more highly connected species has a bigger impact on a network and results in more secondary extinctions. This is comparable to our premise that it is important to identify critical elements in a supply chain that play an important role in maintaining the underlying structure. Understanding the effects of node loss due to perturbations is important also to studies of complex networks such as neural, metabolic, and the World Wide Web [22], [40], [47].

Comparisons across the six real-world examples revealed that the NPF supply chain had the lowest SCI whereas the Sydney rock oyster scored highest (Table 9). The latter was the most linear or streamlined of the supply chain networks. The extremely high SCI_j_ for the two key elements in the oyster supply chain (Figure 5) reinforce the high risk to the stability of this chain if one of these elements is perturbed. However, the wholesale sector in the oyster supply chain is likely to be less affected by disrupted oyster production and interruptions at interim storage and transportation nodes than individual growers. This is because the wholesalers spatially diversify their supply sources in order to spread their business risk. In such interrupted supply situations oyster growers may find it more difficult and costly to establish or re-establish links with supply chain elements further along the chain. The risk of the highly linear supply chain, particularly at the lower end of the chain, is therefore likely to be borne by growers rather than wholesale elements further along the oyster supply chain.

Of the three lobster supply chains, TRL had the lowest SCIT, followed by WRL, with SRL scoring highest. The relatively greater diffuseness of the TRL supply chain is evident too from Figure 5 (and the pie graph shown in the Supporting Information Material (File S1)) which shows a fairly even spread in the individual scores of the top few key elements, compared to a more critical dependence on three key individual supply chain elements for SRL. The SCI score for WRL is lower than for TRL which suggests that it is more diffuse than TRL if the large number of elements (WRL has the most elements and links) in this chain is taken into account. The next logical step in this analysis is to combine all the Australian rock lobster producers into a single chain as two of the fisheries (SRL, WRL) intersect at the same domestic market and the third (TRL) flows into predominantly the same international market (Figures 3, S1, S2 in File S1). Future work will use the SCI applied to this integrated chain to explore the effect of one producer on the other producers.

There was no relationship between the two simple ‘unweighted’ connectance measures, namely links per node and connectance, and the SCI scores (Table 9), highlighting that the new index incorporates additional information. Interestingly, the average connectance measured across the seven supply chain models (0.10) is remarkably similar to mean connectance values for food-webs (approximately 0.11 – [22]). The SCI is easy to interpret given that it is scaled from zero to one, and in the case studies examined there was as much as a six-fold difference between the highest and lowest scores.

Comparison of the distribution of the SCI_j_ across the different stages of the chain suggests some differences between supply chains (Figure 5). For example, key elements for the Sydney rock oyster supply chain are heavily skewed towards the upstream transport and storage, whereas those for the aquaculture prawn example are skewed towards the downstream wholesale and retail and the banana prawn example has a more even spread in terms of the distribution of key elements along the chain. Strictly, the aquaculture examples are not comparable to the other examples presented as they relate to a different sector, but they are included nonetheless as a preliminary example of an application to the farmed rather than wild seafood sector.

Although flexible and holistic in its application, it is important to note that the SCI is only one component of a complete supply chain assessment. Different aspects of each phase of the supply chain have to be considered in further detail. For instance, in the SCI the catch sector (fishers), at the start of the chain, has no numerical impact on the SCI. In reality however, the number and nature of the fishing fleets (fishers) are an important consideration in the context of the efficiency of the supply chain and ultimately in terms of resilience to climate change impacts. If for instance, reduced processor margins from redirecting product from international to domestic markets were passed on to fishers by lowering beach prices, thus squeezing their profit margins, the chain may cross a lower end throughput threshold and the whole chain could potentially collapse. From the fisher perspective, the question then is: at current catch rates, costs and prices, what is the chain I need to see maintain itself to remain in business? Who are the key agents? And what could happen to them if external shocks took place? Moreover, there are inputs to the producers that can also affect the chain (e.g. fuel to boats) but these have not been considered. The SCI also does not take into account the nature of organisations and their (personal, professional and business) relationships within each chain, both of which have strong bearings on the ability of chains to remain resilient and adapt to changes in their environment. However, the SCI provides a perspective that, combined with the understanding of the strengths and weaknesses of relationships in supply chains, can be harnessed to identify opportunities for organisational-level supply chain management.

The SCI is a simple and objective tool for use in sectoral and industry-level economic research. Complementing qualitative analyses, it is a quantitative approach that facilitates standardisation of supply chain sectors, and allows simple interpretation of the index, and the ability to easily identify key elements of supply chains, thereby allowing future scenarios to be objectively evaluated. The ability to holistically consider all supply chain aspects of a sector or industry through the SCI, as done here for different fisheries, will also be of considerable benefit for a range of primary production sectors and will allow comparisons and learning to flow from potential future research.

Additional information would be gained from being able to apply the approach to models of supply chains defined in value terms. In addition, further empirical research on the structure of the costs of adaptation at different levels of the supply chains considered in this study would provide strong grounds for quantitatively assessing the likely ability for Australian wild caught and aquaculture seafood supply chains to adapt to the potential impacts of global changes.

## Supporting Information

File S1 **Details of application to selected Australian supply chains.** This file contains Figure S1-Figure S8. Figure S1, TRL supply chain (after [3]) with colour coding to highlight key elements, with the relative distribution of these summarised in the accompanying pie diagram. Figure S2, WRL supply chain model (from [4]) with colour coding to highlight key elements, with the relative distribution of these summarised in the accompanying pie diagram. Figure S3, Sydney rock oyster supply chain [1] for Queensland and New South Wales, Australia, with colour coding to highlight key elements, with the relative distribution of these summarised in the accompanying pie diagram. Figure S4, Banana prawn (Northern Prawn Fishery) supply chain [1] with colour coding to highlight key elements, with the relative distribution of these summarised in the accompanying pie diagram. Figure S5, Commonwealth trawl supply chain [1] with colour coding to highlight key elements, with the relative distribution of these summarised in the accompanying pie diagram. Figure S6, Aquaculture prawn supply chain (CDI Pinnacle Management 2008) with colour coding to highlight key elements, with the relative distribution of these summarised in the accompanying pie diagram. Figure S7, Pie diagrams summarising the relative distribution of SCI_j_ individual scores for (A) Southern rock lobster, (B) Torres Strait lobster, (C) Western rock lobster, (D) banana prawns, and (E) Commonwealth Trawl Sector. The most critical elements are represented by the larger pie slices, colour coded for all elements with a score that is 1% or more of the total summed score. From highest to lowest scores, the colour coding used is roughly red (>20%)-orange-green-blue-purple. Figure S8, Pie diagrams summarising the relative distribution of SCI_j_ individual scores for two aquaculture examples (A) Sydney rock oysters, and (B) aquaculture prawns. The most critical elements are represented by the larger pie slices, colour coded for all elements with a score that is 1% or more of the total summed score. From highest to lowest scores, the colour coding used is roughly red (>20%)-orange-green-blue-purple.
(PDF)
