# Supplementary material for: A Quantitative Metric to Identify Critical Elements within Seafood Supply Networks
Source: PLoS One. 2014 Mar 14;9(3):e91833. doi: 10.1371/journal.pone.0091833 (PMC3954797; doi:10.1371/journal.pone.0091833)
Supplement: File S1 — Details of application to selected Australian supply chains. This file contains Figure S1-Figure S8. Figure S1, TRL supply chain (after [3]) with colour coding to highlight key elements, with the relative distribution of these summarised in the accompanying pie diagram. Figure S2, WRL supply chain model (from [4]) with colour coding to highlight key elements, with the relative distribution of these summarised in the accompanying pie diagram. Figure S3, Sydney rock oyster supply chain [1] for Queensland and New South Wales, Australia, with colour coding to highlight key elements, with the relative distribution of these summarised in the accompanying pie diagram. Figure S4, Banana prawn (Northern Prawn Fishery) supply chain [1] with colour coding to highlight key elements, with the relative distribution of these summarised in the accompanying pie diagram. Figure S5, Commonwealth trawl supply chain [1] with colour coding to highlight key elements, with the relative distribution of these summarised in the accompanying pie diagram. Figure S6, Aquaculture prawn supply chain (CDI Pinnacle Management 2008) with colour coding to highlight key elements, with the relative distribution of these summarised in the accompanying pie diagram. Figure S7, Pie diagrams summarising the relative distribution of SCIj individual scores for (A) Southern rock lobster, (B) Torres Strait lobster, (C) Western rock lobster, (D) banana prawns, and (E) Commonwealth Trawl Sector. The most critical elements are represented by the larger pie slices, colour coded for all elements with a score that is 1% or more of the total summed score. From highest to lowest scores, the colour coding used is roughly red (>20%)-orange-green-blue-purple. Figure S8, Pie diagrams summarising the relative distribution of SCIj individual scores for two aquaculture examples (A) Sydney rock oysters, and (B) aquaculture prawns. The most critical elements are represented by the larger pie slices, colour coded for all elements [file pone.0091833.s001.pdf]

Supporting Information

A quantitative metric to identify critical elements within seafood supply networks

1. Application to selected Australian supply chains

The SCI approach has been applied to seven Australian supply chain examples: southern rock lobster *Jasus edwardsii* (SRL), Torres Strait tropical rock lobster *Panulirus ornatus* (TRL), western rock lobster (WRL) *P. cygnus*, Sydney rock oysters *Saccostrea glomerata*, Northern Prawn fishery (NPF) banana prawns *Penaeus merguiensis*, Commonwealth trawl and Australian aquaculture prawn supply chain. The first example is described in the main text, and the remainder are briefly described in this Supporting Information. Two examples differ from the set because they relate to farmed seafood rather than wild seafood, and hence although not directly comparable, are included as an illustration of applying the approach to a sector other than wild fisheries. The supply chains for each of these industries were mapped to include the following key economic agents: fishers, interim storage, fish receivers, interim transport, interim storage, primary wholesale, secondary wholesale, export destination, consumers, and hence presented using common templates [1]. Information available on the quantities of fish product flowing between these agents were used to characterize the links between these agents. Mapping the supply chain served as a basis for developing the supply chain models for each industry (Figs S1-S6).

The individual SCI scores for each element in each of the supply chains are ranked and compared to identify critical elements, which have been highlighted in the supply chain schematics and accompanying pie diagrams presented below for wild fisheries (Figure S7) and the two aquaculture examples (Figure S8). The SCI scores are also compared between supply chains, as summarised in Table 1 of the main text.

2. The tropical rock lobster (TRL) supply chain

The Torres Strait tropical rock lobster fishery is the region's economically most important fishery, is a shared stock with Papua New Guinea (PNG), and includes multiple jurisdictions within Australia (the Torres Strait fishery and East Coast fishery, although the same stock, are managed respectively by federal and state agencies). The Australian component of the fishery is exploited by both indigenous Islanders, for whom it has cultural significance, and commercial fishers, most of whom are non-Islanders [2]. TRL are passed down the supply chain either as live lobsters, which are mostly exported to China or frozen tails that are exported to the US, predominantly via a holding facility in Cairns [2].

For TRL, the SCI identified the Chinese and U.S. markets as key elements (Figure S1), suggesting that the key mechanism for stabilising this supply chain is to reduce uncertainty in supplying these markets. Maintaining and strengthening relationships with international markets may thus be the key to underpinning the success of this supply chain. However the individual element scores were not as high as was the case for the key SRL elements, with a more even spread of important elements, suggesting less critical dependence on key elements. This is even more the case if one considers that there is an additional connection or supplier

of product (not considered in this study given logistical constraints), namely the Queensland East Coast lobster fishery which targets the same species, albeit in a different geographical area, and also supplies the Cairns processor.

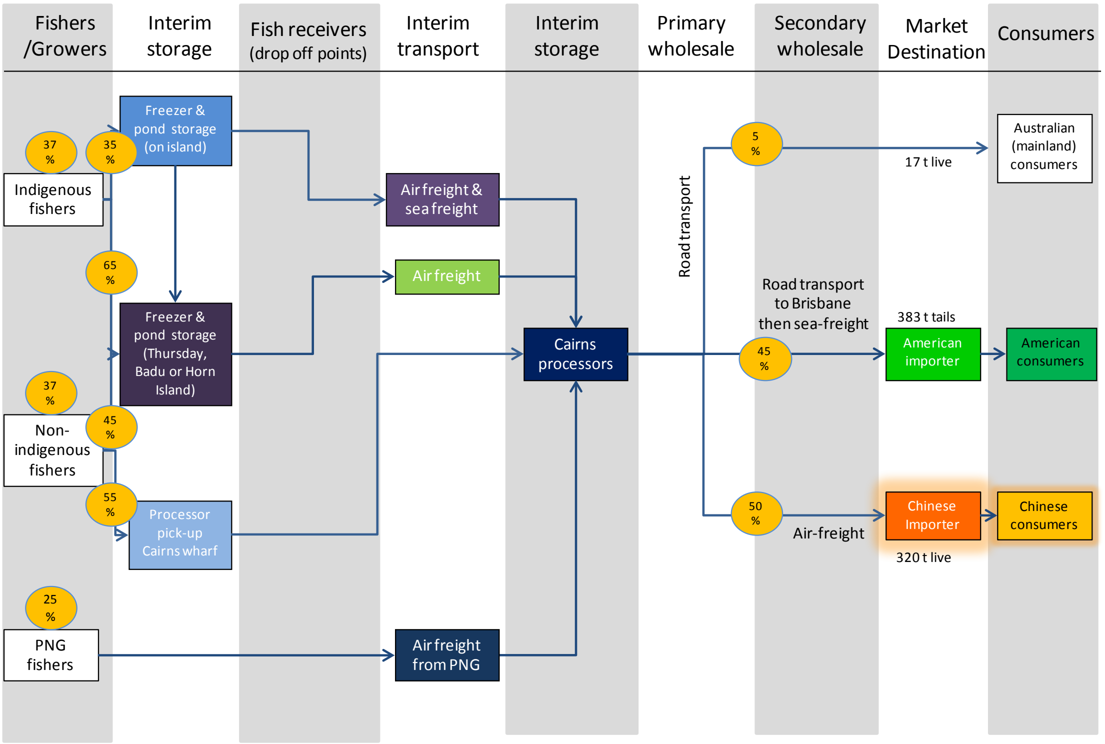

Figure S1. TRL supply chain (after [3]) with colour coding to highlight key elements, with the relative distribution of these summarised in the accompanying pie diagram.

3. The western rock lobster (WRL) supply chain

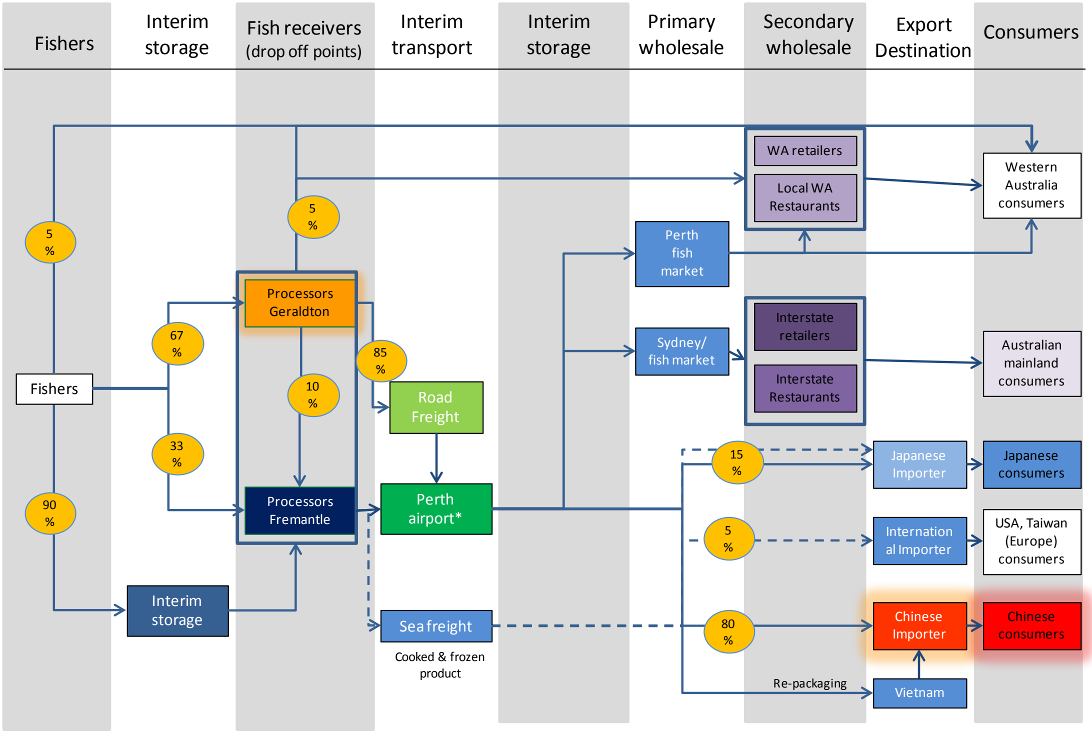

* Australian sales and export figures vary by year

Figure S2. WRL supply chain model (from [4]) with colour coding to highlight key elements, with the relative distribution of these summarised in the accompanying pie diagram.

The WRL fishery is Australia's largest and most valuable lobster fishery. Most of the product is sold in live form and exported to China. Environmental conditions drive variability in this resource inter alia through impacts on puerulus settlement and catchability [5].

The WRL supply chain had a comparatively high number of elements and links (Table 1). The SCI identified the Chinese consumers and associated Hong Kong importer as key elements, followed by the Geraldton processors (Figure S2). The distribution of the individual element scores was more similar to SRL than TRL (Figure 5).

4. Sydney rock oyster

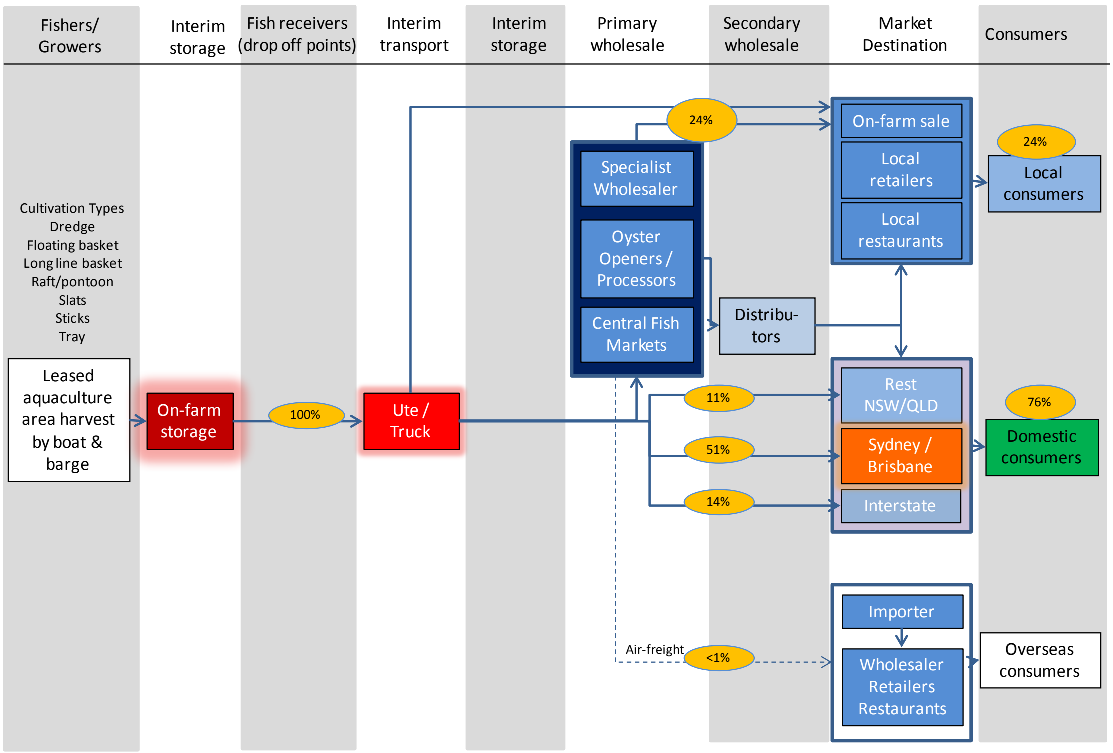

Figure S3. Sydney rock oyster supply chain [1] for Queensland and New South Wales, Australia, with colour coding to highlight key elements, with the relative distribution of these summarised in the accompanying pie diagram.

Sydney rock oysters are cultivated in estuaries along the eastern coast of Australia. Only a small proportion is exported, with the majority marketed locally via on-farm sales, fish mongers, retail stores (namely supermarkets) and restaurants.

The Sydney rock oyster supply chain is highly linear at the supply end, with the interim storage and transport identified as key elements (Figure S3). The dominance of these two elements suggest that this supply chain may be particularly vulnerable to external factors impacting on these key elements, and hence that this chain may not be as resilient as some of the other examples, except if the high degree of integration generates economics of scale in adaptation costs. The Sydney rock oyster example shows a resilience which in contrast to the SRL example above is dependent upon the ability for only two key elements to maintain themselves. However it may simultaneously be an opportunity that growers can take advantage of, knowing the importance of these activities, which they own, to the rest of the supply chain. Without this, a collapse of the entire supply chain and failure to preserve connectivity could occur in the face of threats such as disease affecting the oysters.

5. Banana Prawn (NPF)

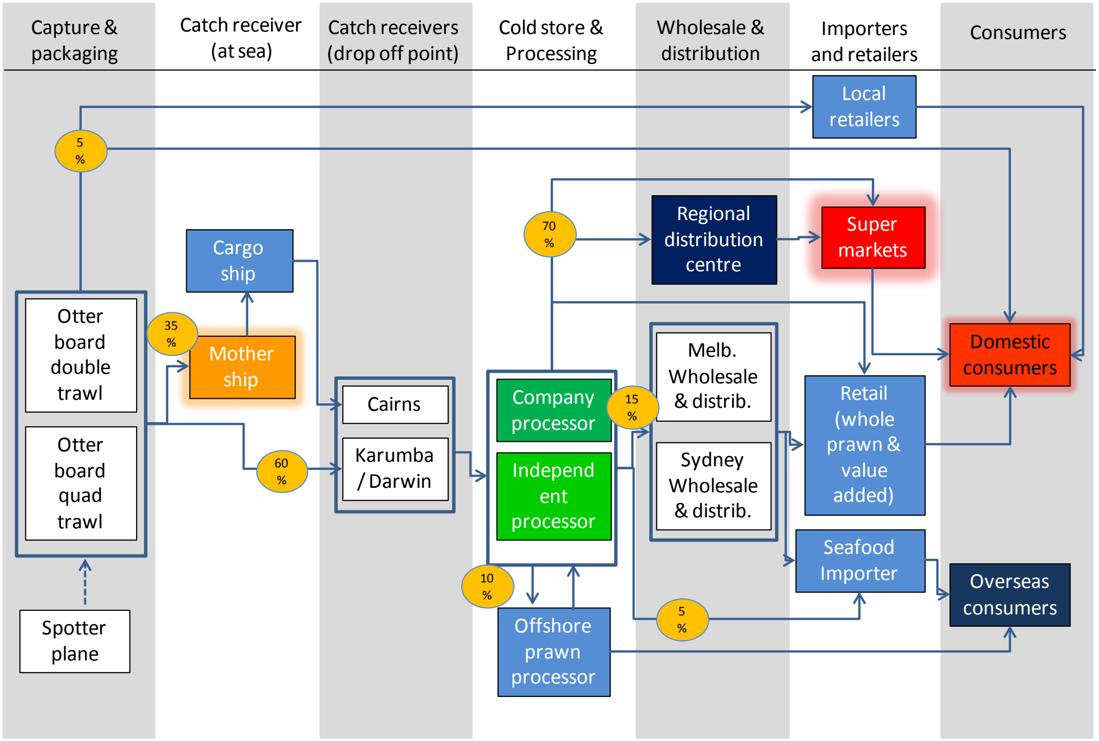

Figure S4. Banana prawn (Northern Prawn Fishery) supply chain [1] with colour coding to highlight key elements, with the relative distribution of these summarised in the accompanying pie diagram.

The Northern Prawn Fishery (NPF) operates in the Gulf of Carpentaria in Australia’s far north, and targets several species of prawns including brown tigers (*Peneaus esculentus)*, grooved tigers (*Peneaus semisulcatus)*, endeavour prawns (*Metapenaeus endeavouri*; a bycatch species) and with the bulk of the revenue obtained from harvesting common banana prawns (*Penaeus merguiensis*) [6]. Production rates of banana prawns are heavily affected by rainfall and can vary considerably from year to year.

For Northern Prawn Fishery banana prawns, the supermarkets and the domestic consumers they supply were identified as key elements (Figure S4). This highlights that it is critical to secure a good working arrangement with the supermarkets. For example, the stability of the supply chain can be improved by focusing effort on determining what factors (e.g. steady supply, minimum volumes of product) are necessary to maintain this as a successful link. In general the banana prawn supply chain showed a relatively good spread of key elements across the chain, and hence an ability to change and adapt connections in response to exogenous shocks.

6. Commonwealth Trawl Sector

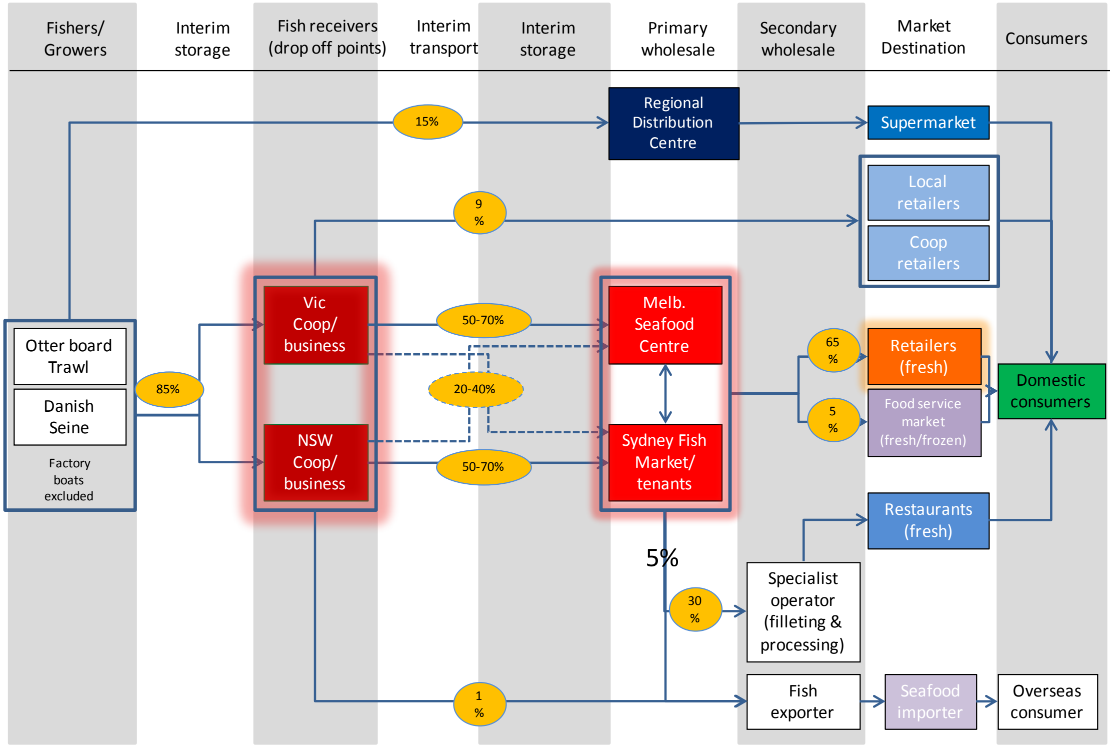

Figure S5. Commonwealth trawl supply chain [1] with colour coding to highlight key elements, with the relative distribution of these summarised in the accompanying pie diagram.

The Commonwealth Trawl Sector (CTS) extends from the waters of southern Queensland to South Australia. It is one of four sectors in the Southern and Eastern Scalefish and Shark Fishery (SESSF), and is the largest sector in catch and value terms, targeted a mixed bag of some 20 species. The majority of the catch is sold to the Sydney and Melbourne Fish markets, and is consumed domestically.

The key elements identified by application of the SCI to the Commonwealth trawl supply chain were the Victoria and New South Wales Cooperatives and businesses and the Melbourne and Sydney markets (Figure S5). The fresh retailers were also identified as important elements in the chain. Of all the chains, this one has critical elements spread across the chain.

7. Aquaculture Prawn Supply Chain

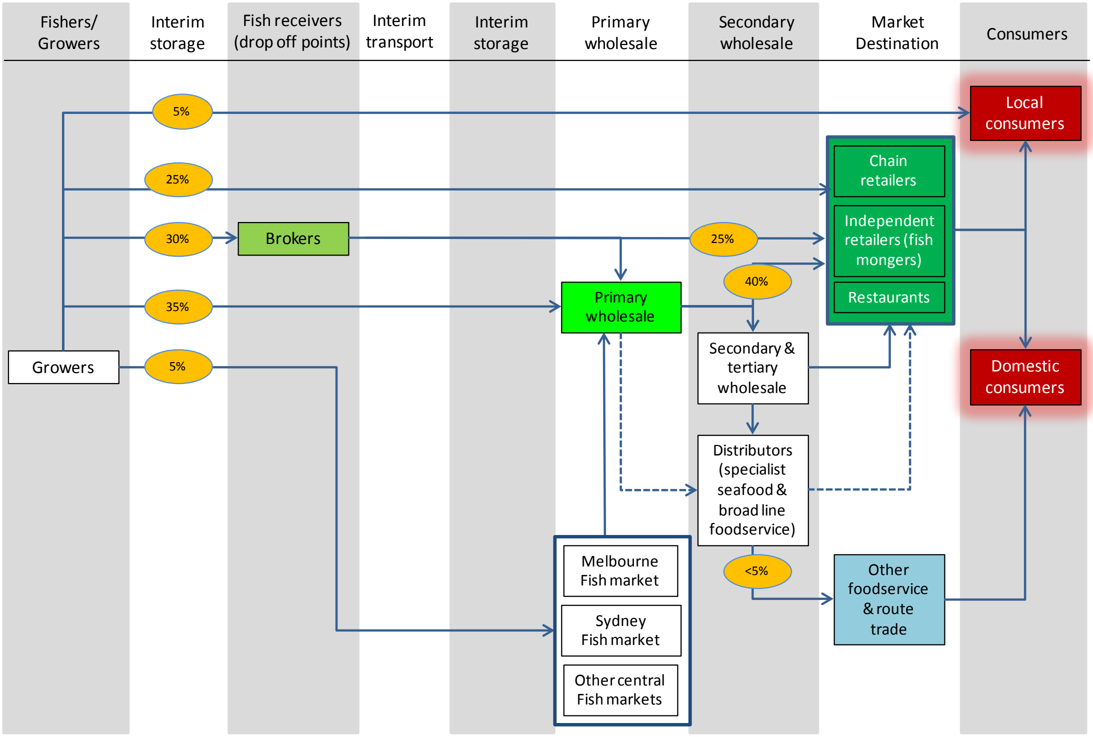

Figure S6. Aquaculture prawn supply chain (CDI Pinnacle Management 2008) with colour coding to highlight key elements, with the relative distribution of these summarised in the accompanying pie diagram.

The aquaculture prawn supply chain differed from the previous examples in that there was a single dominant key element, namely the domestic consumers (Figure S6). However the global structure in this farmed seafood example is different to the other wild seafood examples. Although this case is thus not strictly comparable, it is nonetheless included as an example of an application to a different sector.

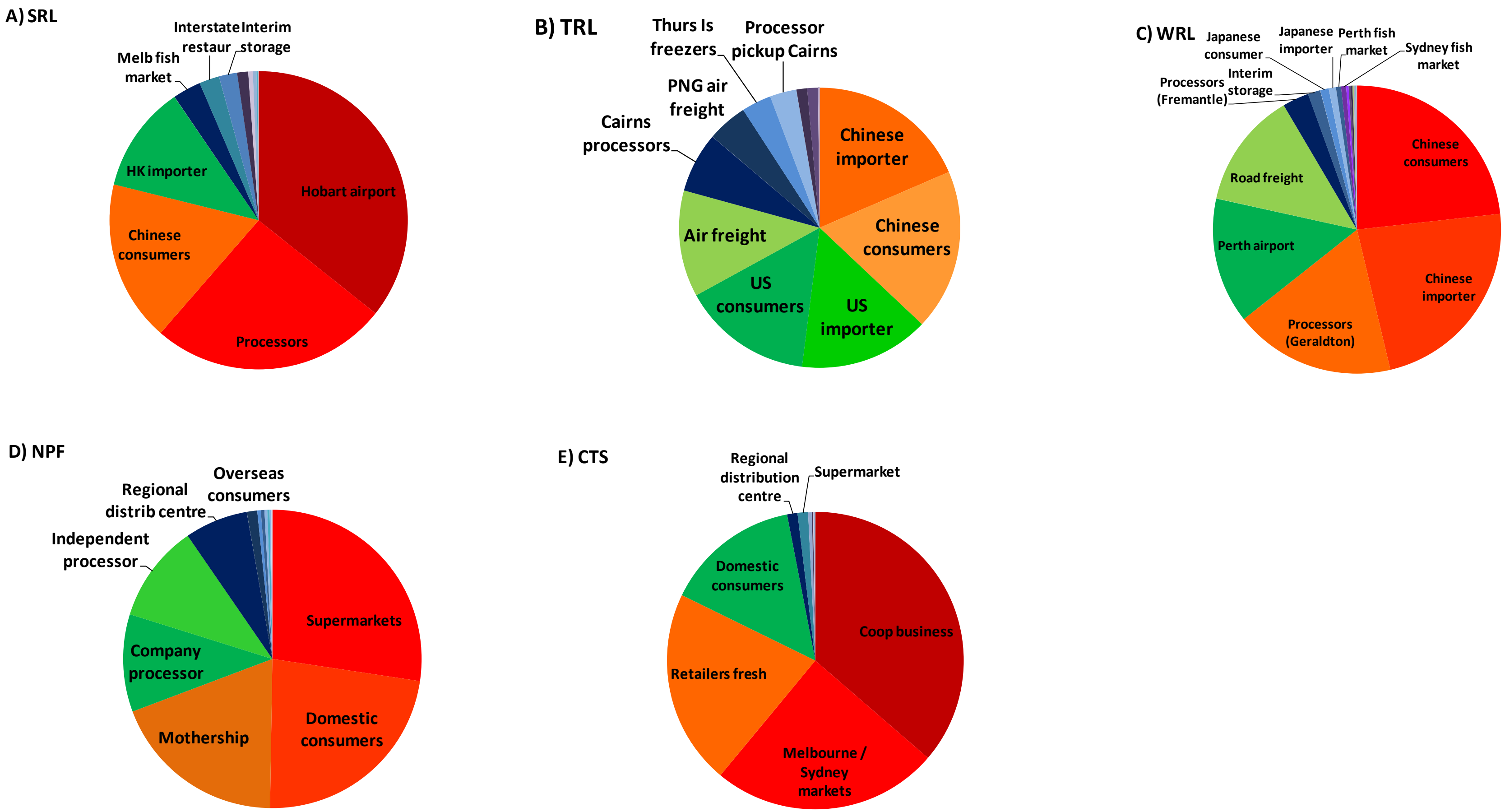

Figure S7. Pie diagrams summarising the relative distribution of SCI_j individual scores for (A) Southern rock lobster, (B) Torres Strait lobster, (C) Western rock lobster, (D) banana prawns, and (E) Commonwealth Trawl Sector. The most critical elements are represented by the larger pie slices, colour coded for all elements with a score that is 1% or more of the total summed score. From highest to lowest scores, the colour coding used is roughly red (>20%)-orange-green-blue-purple.

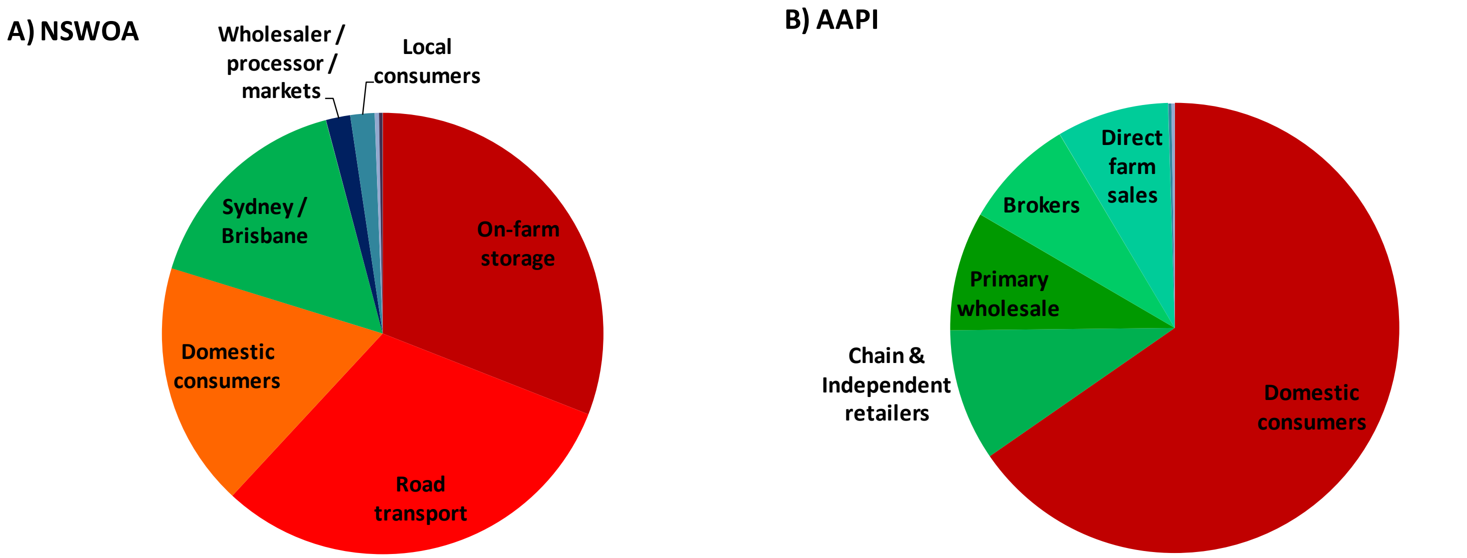

Figure S8. Pie diagrams summarising the relative distribution of SCI_j individual scores for two aquaculture examples (A) Sydney rock oysters, and (B) aquaculture prawns. The most critical elements are represented by the larger pie slices, colour coded for all elements with a score that is 1% or more of the total summed score. From highest to lowest scores, the colour coding used is roughly red (>20%)-orange-green-blue-purple.

References

1. Hobday AJ, Bustamante RH, Farmery A, Fleming A, Frusher S, et al. (2013) Growth opportunities & critical elements in the supply chain for wild fisheries & aquaculture in a changing climate.
2. Plaganyi EE, van Putten I, Hutton T, Deng RA, Dennis D, et al. (2013) Integrating indigenous livelihood and lifestyle objectives in managing a natural resource. Proceedings of the National Academy of Sciences of the United States of America 110: 3639-3644.
3. Plagányi ÉE, Deng R, Dennis D, Hutton T, Pascoe S, et al. (2012) An integrated Management Strategy Evaluation (MSE) for the Torres Strait tropical rock lobster Panulirus ornatus fishery.
4. van Putten IE, A. F, B.S. G, Hobday AJ, L. L-C, et al. (in review) The supply chains of two Australian rock lobster fisheries under a changing climate. .
5. Caputi N, Melville-Smith R, de Lestang S, Pearce A, Feng M (2010) The effect of climate change on the western rock lobster (Panulirus cygnus) fishery of Western Australia. Canadian Journal of Fisheries and Aquatic Sciences 67: 85-96.
6. Dichmont CM, Pascoe S, Kompas T, Punt AE, Deng R (2010) On implementing maximum economic yield in commercial fisheries. Proc Natl Acad Sci U S A 107: 16-21.
